# A quantitative method for estimating the adaptedness in a physiological study

**DOI:** 10.1186/s12976-019-0111-7

**Published:** 2019-09-03

**Authors:** Vladimir N. Melnikov

**Affiliations:** Institute of Physiology and Basic Medicine, P.O. Box 237, 4, Timakov Str, Novosibirsk, 630117 Russia

**Keywords:** Physiological adaptation, Mathematical model, Homeostasis, Plant adaptogens

## Abstract

**Background:**

Existed mathematical models of individual adaptation are mostly reductionist by nature. Researchers usually a priori consider the subject adapted basing only on the fact of continued or prolonged influence of the harmful factor. This paper describes a method that allows assessing the physiological adaptedness to experimental challenges on the basis of holistic approach and quantitative criteria.

**Methods:**

The suggested method comprises simple equations and incorporates into the model an indicator that differentiates functions in regard to their significance for determining physiological adaptedness considered as an outcome of the adaptive process.

**Results:**

The proposed empirical model affords the possibility of comparing subjects in respect to their resistance to several loads. Physiological parameters were differentiated with regard to their significance for assessing adaptedness. Two examples of animal adaptation to exercise after physical training and plant adaptogen administration are considered.

**Conclusion:**

The calculated index of adaptedness is useful in that it replaces wordy descriptions of large tables that reveal alterations in numerous parameters of many subjects under study.

## Background

Adaptation as an active process of responding to challenges and adaptedness as a result of this process mean achieving a positive outcome, i.e. survival and reproduction, in the face of adversity. In a wider sense, it includes behavioral, physiological, structural, and genetic changes upon environmental impacts that are beyond the biologically adequate ranges. Apart from traditional key environmental components (warmth, food, salt, water, microelements), the modern living organisms and especially human beings experience growing need to adjust their millieu to arising amount and variability of new materials, drugs, and chemicals. It is just the physiological adaptation that involves ‘active resistance’ and provides achieving that positive outcome, living without diseases, if and when the preceding behavioral adaptation occurs ineffective or insufficient.

Adaptability as a fundamental property of living matter is widely studied by experimental biology and medicine. Every specialist in the field encounters difficulties in selecting criteria of adaptedness and analysing the results of multifactor experiments, wherein the state of the subject is assessed by a set of numerous parameters. Many researches a priori consider the investigated subject adapted to the given factor only on the basis of its prolonged influence.

There have been some previous attempts to quantify adaptedness [1]. Most of them deal with Darwinian adaptation considered as the biological fitness in the context of reproductive success; some relate with pharmacological tolerance as a result of adaptive process [2, 3]. Researches consider adaptation in terms of tolerance, stability, constancy, resistance, coping, in view of stress conception [4]. An analysis of the existed literature reveals that authors stretch the meaning of the term and interpret it loosely from genes and millisecond time scale [5] to such long-lasting weather-related health outcome as cardiovascular mortality [6]. Most studies focus on distinct systems or functions: neural networks [7], visual perception [8], blood circulation [9], carbon dioxide transport [10], physical performance [11, 12]. Pries and co-authors [13] have proposed a mathematical model to explain how a complex interaction of various stimuli can lead to vascular adaptation. Gorban et al. [14] have designed an algorithm based on the number and extent of correlations between parameters characterising the state of the population under study.

The existed models are mostly reductionistic by nature. The empirical model proposed here is built on an organism level and based upon the holistic approach introducing quantitative criteria of individual adaptation. This study is the first to incorporate into the model an indicator that differentiates functions with regards to their significance for determining physiological adaptedness considered as an outcome of the adaptive process.

## Description of the method

In order to estimate the degree of adaptedness to given harmful factor, probably the most direct way is to subject the organism to the action of said factor, applying functional “resolving” or “provoking” loads which force the physiological system to reveal its adaptive possibilities, for example, attained in the course of training or pharmacological pre-treatment. The proposed method, involving simple mathematical operations, makes it possible to obtain an index of adaptedness of bio systems to various environmental or experimental conditions.

### CASE I: two organisms, repeated-measure design

**Fig. 1:**
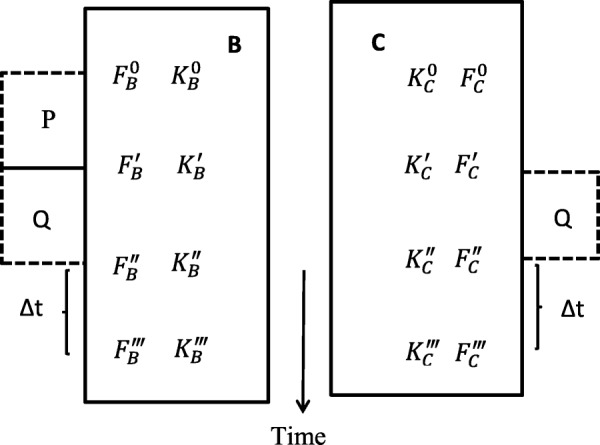
Scheme of the physiological experiment appropriate for the model described

Let us assume that we have two organisms *B* and *C* chosen at random from a homogenous population. Suppose that the individual *B* was subjected to *P*, a disturbing effect of environment or internal stressor. Let us then try to answer the question how the effect of *P* influenced the resistance of *B* to the short-term action of another factor, say *Q*, to which both *B* and *C* are equally subjected for the comparison in an experiment with parallel design (Fig. 1). As a rule, *P* precedes the action of *Q* (not necessarily, however) and is accompanied by a change in the resistance of the organism to *Q*. Qualitatively, both effects may be of the same nature. Thus, for instance, this is observed in effect of prolonged moderate physical training on the tolerance of animals to muscular load or the effect of preventive introduction of poison on the resistance of organisms to subsequent acute poisoning. Yet, the above two effects may be different in nature, this being the case in the study of the effects of pharmacological preparations, pretreatment or preconditioning (*P*) on adaptation to hypoxia, hypothermia, and so on (*Q*).

Out of a multitude of possible situations, let us take the following four states of the subjects studied: (1) baseline intact state prior to the action of *P* (*F*^0^); (2) state subsequent to *P* and prior to *Q* (*F′*); (3) state followed immediately after *Q* (*F*^″^); and (4) state following the lapse of time ∆t after the termination of action of *Q* (*F‴*). It is clear that the first and second states for subject *C* coincide: ${F}_C^{0\kern0.5em }$ *=* ${F}_C^{\prime \kern0.5em }$ (*P* is absent). ${F}_B^{0\kern0.5em }$ *=* ${F}_C^{0\kern0.5em }$ is also justifiable.

The entire set of physiological parameters reflecting the state of the bio system during the action of a given threatening factor may be tentatively divided into at least three categories [15]. The first one includes parameters, mainly homeostatic [16], which primarily and directly change as a result of the action of an entropic agent: body temperature at cooling or overheating, blood oxygen saturation during hypo- or hyperoxia, concentration of lactic acid and content of energetic resources (hepatic and muscle glycogen, glucose, FFA, ATP levels) during physical exercise, etc. The second category includes parameters which reflect changes in adaptive, or allostatic [16], functions and mechanisms, whose work is directed to normalize the initially changed characters, to counter the adverse effects of entropic factor, and to level off shifts in homeostatic variables [17–19]. As in the cases of hypoxia [20] and physical load [12], such parameters would be heart and respiration rates, cardiac output, secretion of adaptive hormones corticosteroids and catecholamines [21], etc. Variables of the third category do not change at all under the above-said action. In other words, they characterize functions indifferent to the influencing factor.

The boundary between the first and second groups is sometimes tentative. It is clear that the values of some functions may sometimes alter with environmental changes in the manner depending on the severity and timing of stress: from initially reacting mechanisms they may become pronouncedly allostatic and vice versa. Nevertheless, it is usually possible to establish such a boundary in each concrete situation from the physiological view.

Changes in the parameters of the homeostatic and adaptive categories under the effect of a given factor should be accounted for with opposing signs in assessing the adaptedness of an organism to the said factor. Despite the continuing action of the perturbation agent, the initially changed physiological characters become normalized or less pronounced in the state of adaptation. Contrariwise, the adaptive mechanisms work with greater intensity.

The latter premise has been suggested as a result of an analysis of experiments performed in different studies. Thus, for instance, it is known that trained athletes in response to intensive physical loads (*F*″) show higher cortisol secretion [22] mainly due to the decrease in post-training value (*F′*) of basal secretion [23, 24]. Untrained people, however, did not display such increased secretion, or even showed a decline thereof. Cases of normalization of a given adaptive function in the process of adaptation, observed by many investigators, indicates a “redistribution of roles” among functions, when a less powerful mechanism had exhausted its possibilities and has been replaced by another more powerful and stable one, a mechanism that rarely “actuates” (in states remote from exhaustion).

Let us assess the state of tested organisms by the set of parameters *K*_*1*_, *K*_*2*_*,…, K*_*j*_*, …, K*_*m*_, which, in our view, are the most important in estimating adaptedness to *Q*. Theoretically, a situation may arise wherein out of the entire set, we will choose for investigation such parameters that do not change under the action of *Q*; then *F′* = *F″*. In practice, however, this is almost improbable, since the investigator usually has sufficient information as to what characteristics should change under *Q*. The difference *K″ – K′* characterizes a change in *K* under the action of *Q*. Since the responding variables can change in either positive or negative direction from the baseline, their reactions should be considered as a modulus. In order to have a possibility to compare such differences irrespective of the sign and absolute value of the parameter, we will further on use the relative magnitude ∣*K*^″^ – *K* ′  ∣ /*K*′.

To assess recovery process during the rest of the characteristic that had changed as a result of the action of *Q*, let us consider the fraction ∣*K*^‴^ – *K* ′  ∣ / ∣ *K*^‴^ – *K* ″ ∣. The numerator shows how close the changed parameter had “returned” to its initial value prior to the action of *Q*. In view of the present model, a lesser difference indicates better tolerance. The physiological practice indicates that the fraction is not sensitive to K′, so the possible effect of the difference between *K’*_*B*_ and *K’*_*C*_ for a given variable may be ignored in such an approximate algorithm. The difference *K‴ – K″* characterizes the rate of parameter change during a given period of time *∆t*. It is suggested that the higher the rate of recovery and the greater the difference *K‴ – K″*, the more adaptive is the organism. Precisely for that reason, the rate index is shown in the denominator in order to minimize the fraction

1 $$ \mid {K}^{{\prime\prime\prime} }-K^{\prime}\mid /\mid {K}^{{\prime\prime\prime} }-K^{{\prime\prime}}\mid \to \min, $$

when approaching the state of adaptation, wherein vitally important constants are kept on a normal level or, in other words, when stability of the usual level of specific vital activity, viability and reproducibility of the bio system is maintained under conditions of continuing action of a stressor. In this case, we stem from the following criteria of adaptedness: an organism adapted to *Q* responds to the action of it with lesser shifts in homeostasis, lesser deviations from “tranquil” values of the initially changing parameters during more intensive work of adaptive mechanisms. Secondly, during the state of adaptation, the process of recover of indices that changed under *Q* becomes accelerated. These criteria may be expressed mathematically as follows:

$$ \left\{\begin{array}{c}\frac{\mid {K}^{{\prime\prime} }-{K}^{\prime}\mid }{K^{\prime }}\kern0.5em \to \min, \mathrm{for}\ \mathrm{the}\ \mathrm{primarily}\ \mathrm{changing}\ \mathrm{homeostatic}\ \mathrm{variable};\\ {}\frac{\mid {K}^{{\prime\prime} }-{K}^{\prime}\mid }{K^{\prime }}\kern0.5em \to \max, \mathrm{for}\ \mathrm{the}\ \mathrm{variable}\ \mathrm{representing}\ \mathrm{the}\ \mathrm{adaptive}\ \mathrm{function}/\mathrm{process};\\ {}\frac{\mid {K}^{{\prime\prime\prime} }-{K}^{\prime}\mid }{\mid {K}^{{\prime\prime\prime} }-{K}^{{\prime\prime}}\mid}\kern0.5em \to \min, \mathrm{for}\ \mathrm{the}\ \mathrm{recovery}\ \mathrm{process};\end{array}\right. $$

or for many parameters

2 $$ \left\{\begin{array}{c}{\sum}_{j=1}^m\frac{K_j^{{\prime\prime} }-{K}_j^{\prime }}{K_j^{\prime }}\to \min, \\ {}{\sum}_{h=1}^d\frac{K_h^{{\prime\prime} }-{K}_h^{\prime }}{K_h^{\prime }}\to \max, \\ {}{\sum}_{j=1}^m\frac{\mid {K}_j^{{\prime\prime\prime} }-{K}_j^{\prime}\mid }{\mid {K}_j^{{\prime\prime\prime} }-{K}_j^{{\prime\prime}}\mid }+{\sum}_{h=1}^d\frac{\mid {K}_h^{{\prime\prime\prime} }-{K}_h^{\prime}\mid }{\mid {K}_h^{{\prime\prime\prime} }-{K}_h^{{\prime\prime}}\mid}\to \min, \end{array}\right. $$

on approaching the state of physiological adaptation to *Q*.

Here *j* is the index of directly and primarily changing parameter (*j* = 1, 2, …, *m*); and *h* the index of the variable representing the work of adaptive allostatic functions (*h* = 1, 2,…, *d*). Naturally, all the differences should be within the limits of physiological adequateness of reactions.

The index of the adaptedness of subject *B* to the action of *Q* is:

3 $$ {a}_{B=}{\sum}_{j=1}^m\left(-\frac{\mid {K}_{Bj}^{{\prime\prime} }-{K}_{Bj}^{\prime}\mid }{K_{Bj}^{\prime }}-\frac{\mid {K}_{Bj}^{{\prime\prime\prime} }-{K}_{Bj}^{\prime}\mid }{\mid {K}_{Bj}^{{\prime\prime\prime} }-{K}_{Bj}^{{\prime\prime}}\mid}\right)+{\sum}_{h=1}^d\left(\frac{\mid {K}_{Bh}^{{\prime\prime} }-{K}_{Bh}^{\prime}\mid }{K_{Bh}^{\prime }}-\frac{\mid {K}_{Bh}^{{\prime\prime\prime} }-{K}_{Bh}^{\prime}\mid }{\mid {K}_{Bh}^{{\prime\prime\prime} }-{K}_{Bh}^{{\prime\prime}}\mid}\right). $$

The formula for *a*_*c*_ is the same. It should be noted that the equalities *m*_*в*_ *= m*_*c,*_ *d*_*в*_ *= d*_*c*_ must be maintained. Otherwise, the difference between *a*_*в*_ and *a*_*c*_ will depend upon the differing numbers of the parameters tested since the *a* index is calculated as a sum.

In case *a*_*в*_ > *a*_*c*_, there are grounds, on the basis of the above criteria, to say that *B* is more adaptive to *Q*, than *C*. In case of a contrary inequality, the conclusion should be made that *P* had an unfavorable effect on *B*, this being revealed in a lower resistance of *B* to *Q*.

The magnitude of (1) essentially depends on ∆t. When ∆t is small, K‴ is close to K″, and the difference K‴ – K′ may be large. In this case, ∣ *K*^‴^ – *K* ′  ∣ / ∣ *K*^‴^ – *K* ″  ∣  >  > 1. Such situations should be avoided as they are difficult to analyse by means of the method suggested, since the above fraction would essentially affect the index of adaptedness. It should be noted that in formula (2) the terms of ∣*K*^″^ – *K* ′  ∣ /*K*′ are usually close to unity or comprise its fractions.

To avoid the above-said difficulty, one can assume two solutions. First, to divide the fraction by a constant number, performing this operation for the given parameter K_i_ for all the subjects compared. One may be to a certain extent subjective in selecting the constant number, and this would put K_i_ in an unequivalent position compared with that of the other parameters. Hence, it would be desirable to select such a duration of rest period that would render K‴ significantly different from K″ and close to K′, while the fraction of (1) would be less than unity.

**Fig. 2:**
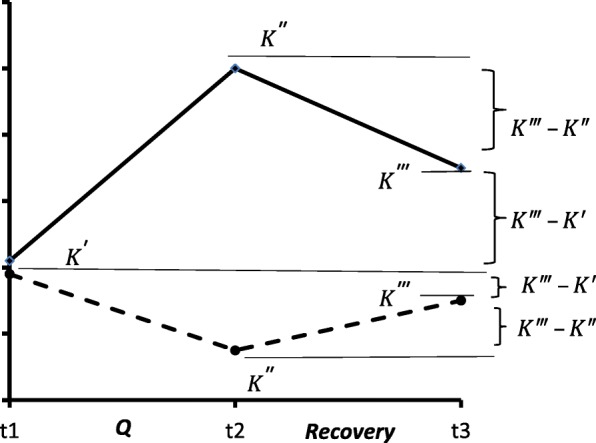
Two different variants of recovery dynamics for more adapted (dotted line) and less adapted (solid line) organisms

At the same time, the interval ∆t = t3 – t2 (Fig. 2) should not be too great (usually from several dozen minutes to several hours), since, during a prolonged rest period, one may become “involved” in a phase of supercompensation of the investigated parameter, when its value would, for a second time, differ from the baseline level. This primarily concerns indices that characterize certain reserves or resources of the organism, which become exhausted under the effect of *Q*.

It would be desirable that *t*_*в*_ *= t*_*c*_. Only in that case there would be no need to introduce ∆t into formulae (1) and (2). Moreover, this would exclude the influence of a possible nonlinearity in time of the process of recovery, the said nonlinearity being dependent on *P*.

Special consideration should be given to cases when the sign, representing an alteration in a parameter under test load *Q* (*K*″– *K*′), changes to the opposite, depending on whether or not *P* affected the organism. Thus, for instance, in the subject *C* the above variable increases under the functional load (${K}_C^{{\prime\prime} }$ > ${K}_C^{\prime }$, *K″ – K′* > 0), while under the same load the organism B responds by a decreased parameter (${K}_B^{{\prime\prime} }$ < ${K}_B^{\prime }$, *K″ – K′* < 0). Such situations are rare, but they are interesting from the theoretical point of view and afford much information on the mechanisms of adaptive reactions.

Let us now assume that, as a result of action of *P*, the mechanism responsible for maintaining *K*_*i*_ within its norms has “exhausted” its possibilities, and the value of the index is either on its possible upper (lower) boundary or has returned to its initial level, but has not correspondingly increased (decreased) to the action of *Q*. At the same time, an organism unaffected by *P* possesses a large reserve for changing the above index up to one its boundaries on the background of unchanged reserves. In this case, *P* has probably had an adverse effect on the system, violating the natural course of the response to the action of *Q*. In all cases, “paradoxical” alterations with the *p* value > 0.05, i.e. statistically negligible, should ostensibly be considered insignificant and taken for zero.

In this connection, the greater the number of parameters considered, the lesser the possibility of an erroneous conclusion, because, in a vast amount of data, an individual, possibly a chance anomalous alteration, of a given parameter, is leveled off to a greater degree.

Let us introduce into (2) a coefficient that characterizes the importance of each parameter for assessing adaptedness. As a criterion of importance, let us take *S*, the stability index calculated by subtracting the variability coefficient from unity: *S*_*x*_ = 1 – $\mid \mathrm{SD}\mid /\overline{K_x^0}$. Here SD is the standard deviation, and $\overline{K_x^o}$ the arithmetical mean of the variational series, comprising the values of the given parameter *K*_*x*_ for a group of intact animals, whose state corresponds to *F*^*o*^:

$$ \overline{K_x^o}=\frac{1}{l}\kern0.62em {\sum}_{y=1}^l{K}_{xy}^o, $$

where *x* is the index of the parameter (established fixed), *x* = 1, 2,…, (*m + d*), and *y* – the index of individual, (*y* = 1, 2, …, *l*).

The real minimum value of coefficient *S* is practically estimated at 0.5, i.e. the statistical distribution of the random magnitude ${K}_x^0$ in a population of intact individuals should approach to normal. Cases when *S* → 0 or *SD ≈* Mean, characteristic of the Poisson and asymmetrical distributions, are not considered by this model. To estimate *S* more accurately, it should be calculated for a sufficiently numerous sampling population of intact individuals. The coefficient *S* depends on *SD* and shows the degree of variability of a given parameter in a group of subjects. It is obvious that constant parameters of the internal medium (*pH*, osmotic blood pressure, body temperature in homeotherms, etc.) have low standard deviation values and *S* values close to unity.

Let us now consider how to take account of the degree of stability of an initially changing parameter when assessing its importance for estimating adaptedness. Considerable alterations in ultrastable parameters under the action of *Q* indicates a low resistance of the organism to *Q*. Contrariwise, an organism adapted to *Q* responds to the latter by changes in characters of low stability, while no or insignificant changes take place in stable and ultrastable constants. Hence, ultrastable characteristics are more important for assessing adaptedness than labile ones. To cite an example, let us assume that in a given organism, subjected to the action of *Q*, the parameter with low stability (*S* = 0.7) changed by 5 units, and the ultrastable one (*S* = 0.9) by 10 units. Contrariwise, in another organism the low stability and ultrastability characters changed by 10 and 5 units, respectively. Then, a_1_ = 5 + 10 = 15, a_2_ = 10 + 5 = 15, and a_1_ = a_2_. Introduction into the formula of a corresponding stability coefficient before each of the parameters would change the equations as follows: a_1_ = 0.7 × 5 + 0.9 × 10 = 12.5; a_2_ = 0.7 × 10 + 0.9 × 5 = 11.5, and a_2_ < a_1_. This, in turn, would allow to make a correct conclusion that the first organism is more adapted to Q.

Argumentation concerning adaptive function should bear a different nature. The labile functions, which are characterized by initially intensive work, but are energetically disadvantageous and possess low “adaptation capability”, are later gradually replaced by deep, highly stable functions, which afford the organism substantial gains in the process of adaptation. Thus, people adapted to hypoxia have respiration and heart rates almost close to normal; at the same time, the changes taking place in their tissues and cells remain to be substantial [25]. Hence, it would be considered preferable to maintain that adaptation takes place on account of ultrastable and deep adaptive mechanisms. In the process of adaptation, there is a possibility of “normalization” of a “low-powered” adaptive mechanism with replacement of its functions by another more stable phylogenetically older and ontogenetically earlier mechanism. Taking this into consideration, all experiments should be planned in such a way as to have the possibility to measure the indices of at least three adaptive functions, which differ considerably with regard to their stability coefficients.

Considering the terms of the recovery process, it should be said that quicker restoration of highly stable parameters to the initial level during rest indicates their better adaptedness. In this case, too, ultrastable parameters are more important for our purpose than low-stability parameters. Accounting for the above-cited considerations, subsequent to the introduction of coefficient *S*, which differentiated all characters with respect to their importance for assessing adaptedness, formula (3) for the integral index would be expressed as:

4 $$ {a}_{B=}{\sum}_{j=1}^m{S}_j\left(-\frac{\mid {K}_{Bj}^{{\prime\prime} }-{K}_{Bj}^{\prime}\mid }{K_{Bj}^{\prime }}-\frac{\mid {K}_{Bj}^{{\prime\prime\prime} }-{K}_{Bj}^{\prime}\mid }{\mid {K}_{Bj}^{{\prime\prime\prime} }-{K}_{Bj}^{{\prime\prime}}\mid}\right)+{\sum}_{h=1}^d{S}_h\left(\frac{\mid {K}_{Bh}^{{\prime\prime} }-{K}_{Bh}^{\prime}\mid }{K_{Bh}^{\prime }}-\frac{\mid {K}_{Bh}^{{\prime\prime\prime} }-{K}_{Bh}^{\prime}\mid }{\mid {K}_{Bh}^{{\prime\prime\prime} }-{K}_{Bh}^{{\prime\prime}}\mid}\right). $$

### CASE II: two groups of organisms, repeated measure parallel experimental design

Let us now consider the case when two groups of subjects numbering n_в_ and n_с_ and selected at random from a qualitatively homogenous population are subjected to the effects of *P* and *Q*. Let us assume that, as in the first case with two organisms, concrete experimental techniques allow us to record the necessary parameters for each subject in four (or three for group *C*) of the above said states (*F*^*o*^*, F*^*′*^*, F*″*, F* ‴), the technique of measuring the characteristics not affecting the state of the subject.

Then, having composed and classically treated variational series based on calculated individual magnitudes *a*_*i*_ and *a*_*r*_, one can obtain the mean values

5 $$ \left\{\begin{array}{l}{\mathrm{A}}_{\mathrm{B}}=\overline{a_{\mathrm{B}}}=\frac{1}{{\mathrm{n}}_{\mathrm{B}}}\;{\sum \limits}_{i=1}^{{\mathrm{n}}_{\mathrm{B}}}{a}_i=\\ {}=\frac{1}{{\mathrm{n}}_{\mathrm{B}}}{\sum \limits}_{i=1}^{{\mathrm{n}}_{\mathrm{B}}}\left[{\sum \limits}_{j=1}^m{S}_j\left(-\frac{\left|{Kij}^{\hbox{'}\hbox{'}\hbox{'}}-{Kij}^{\hbox{'}}\right|}{\left|{Kij}^{\hbox{'}\hbox{'}\hbox{'}}-{Kij}^{\hbox{'}\hbox{'}}\right|}-\frac{\left|{Kij}^{\hbox{'}\hbox{'}}-{Kij}^{\hbox{'}}\right|}{Kij^{\hbox{'}}}\ \right)+{\sum \limits}_{h=1}^d{S}_h\left(-\frac{\left|{K}_{ih}^{\hbox{'}\hbox{'}\hbox{'}}-{K}_{ih}^{\hbox{'}}\right|}{\left|{K}_{ih}^{\hbox{'}\hbox{'}\hbox{'}}-{K}_{ih}^{\hbox{'}\hbox{'}}\right|}++\frac{\left|{K}_{ih}^{\hbox{'}\hbox{'}}-{K}_{ih}^{\hbox{'}}\right|}{K_{ih}^{\hbox{'}}}\ \right)\ \right]\\ {}{A}_C=\overline{a_c}=\frac{1}{{\mathrm{n}}_c}{\sum \limits}_{r=1}^{{\mathrm{n}}_c}{a}_r\end{array}\right. $$

and standard deviations, make up confidence intervals, and estimate the authenticity of the difference between *A*_*B*_ and *A*_*C*_ with any pre-estimated probability of error. In formulae (5), *i* is the index of the individual in group *B*, *r* the index of an organism in group *C*, *j* the index of a directly changing homeostatic parameter, and *h* the parameter index of allostatic functions. Multipliers 1/*n*_*B*_ and 1/*n*_*C*_ may be omitted in formulae if *n*_*B*_ = *n*_*C*_.

### CASE III: parallel design

The next design is widely distributed in experimental biology and physiology, where the imperfection of investigation techniques or their specific properties often lead to the necessity of killing the animal in order to obtain certain characteristics of its internal medium. This is justified for almost all morphological and biochemical investigation methods with the exception of cases involving biological fluids. In this case, several animals are killed in each *F* state, and, on the basis of their individual properties, the mean value for each parameter (in case of need, its SD also) is estimated:

6 $$ \overline{K_t^{\hbox{'}}} = \frac{1}{g}\;{\sum}_{f=1}^g{K}_{tf}^{\hbox{'}}, $$

where *f* is the index of individual, *g* the index of individuals used for obtaining parameter *t* in state *F*′, *t* the parameter index, t = 1, 2,…, (m + d). Accounting for this important circumstance, each parameter in (4) should be replaced by its mean value (6).

In case III, it is more difficult to calculate the standard deviation for the index of adaptedness than in Case II, even though there are mean error for each parameter in each of the states. The problem is to calculate the standard variation of the sum of (4) with corresponding replacement of K by $\overline{K}$ in accord with (6).

## Example 1

### Materials and methods

Let us consider, for example, the calculation of the index of adaptedness for several groups of animals. This case may be assigned to the last and, apparently, the most complex of the algorithm described. The experiment was performed by the author in collaboration with Drs. A.V. Shulga, E. Khasina, and G. Bezdetko. The numerical application of the method is presented in Tables 1 and 2.

**Table 1:** Biochemical parameters in rats subjected to physical training, eleutherosides administration, and acute exercise

Parameter	S	Control				Injections of eleutherosides			
		Untrained (B)		Trained (C)		Untrained (D)		Trained (E)	
		Rest (*N* = 10) F ° = F ′	Swimming (*N* = 10) F ″	Rest (*N* = 8) F ′	Swimming (*N* = 9) F ″	Rest (*N* = 9) F ′	Swimming (*N* = 9) F ″	Rest (*N* = 8) F ′	Swimming (*N* = 8) F ″
Gastrocnemius muscle hexokinase activity, nM NADPH × mg protein^−1^ × min^− 1^	0.913^a^	13.8 ± 1.2	7.1 ± 1.7	13.4 ± 1.7	12.7 ± 2.0	14.2 ± 1.1	5.5 ± 2.3	13.1 ± 2.8	8.6 ± 1.4
		0.485 ^b^		0.052		0.600		0.344	
Blood ammonia, μg × 100 mL^−1^	0.893	103 ± 11	186 ± 48	83 ± 18	127 ± 73	79 ± 13	110 ± 34	73 ± 18	112 ± 51
		0.805		0.530		0.393		0.535	
Plasma 11-HCS, μg × 100 mL^−1^	0.778	13.1 ± 2.9	30.8 ± 6.8	8.1 ± 2.9	30.2 ± 5.7	8.9 ± 1.2	31.9 ± 2.6	10.9 ± 1.5	28.8 ± 2.5
		1.35		2.73		2.59		1.64	
Muscle glycogen, mg × 100 g^−1^	0.694	702 ± 215	293 ± 218	897 ± 269	451 ± 147	745 ± 173	225 ± 124	757 ± 165	387 ± 153
		0.580		0.500		0.700		0.490	
Blood lactate, mmol ×L^− 1^	0.672	6.1 ± 2.0	16.6 ± 8.5	5.5 ± 0.8	7.2 ± 1.4	5.2 ± 1.1	12.5 ± 6.2	5.5 ± 1.1	9.8 ± 7.4
		1.720		0.310		1.400		0.780	

*11-HCS* 11-hydroxycorticosteroids; ^a^ 0.913 = 1–1.2 / 13.8; ^b^ 0.485 = |7.1–13.8| / 13.8 = |K″ – K′| / K′. Data are expressed as Mean ± SD

**Table 2:** Calculation of the index of rat adaptedness to short-term intensive muscular work

Parameter	Control		Introduction of eleutherosides	
	Untrained, B	Trained, C	Untrained, D	Trained, E
11-HCS	+ 1.060	+ 2.120	+ 2.010	+ 1.273
Hexokinase	− 0.445	−0.048	− 0.500	−0.315
Lactate	− 1.163	−0.209	− 0.946	− 0.527
Ammonia	− 0.715	− 0.473	− 0.350	− 0.473
Glycogen	−0.402	− 0.347	− 0.486	− 0.340
$A=\sum S\frac{\mid {K}^{{\prime\prime} }-{K}^{\hbox{'}}\mid }{K^{\hbox{'}}}$	− 1.665	+ 1.043	− 0.272	− 0.382
Adaptive rank	4	1	2	3

The index was computed per formula (4) with corresponding substitution of (6). Terms characterizing normalisation of parameters during recovery are absent

Sexually mature male Wistar rats from groups *D* and *E* (Table 1) were subcutaneously injected with 0.5% aqueous solution of a sum of eleutherosides (active substances from the roots of the Far Eastern plant *Eleutherococcus senticosus*). This remedy like ginseng belongs to adaptogenic herbs [26, 27] that are known to hold a capacity to increase stress resistance, physical and mental performance without increasing oxygen consumption [28]. The injections were made in dose of 5 mg/kg twice a day for fourteen days. Animals from groups *C* and *E* were trained daily for a fortnight by swimming with a 6% load on the tail. The initial session was 12 min, plus 2 min every subsequent day. Animals from group C were injected with an isotonic solution of sodium chloride. The animals were killed 46 h. after completing the last swimming session. Immediately before that, they were subjected to a functional load involving 15-min swimming with 6% load in water, temperature 29–31 °C. Some of the rats were decapitated prior to swimming (state of rest).

### Results

The results of the experiment and their treatment for obtaining the index of adaptedness are presented in Tables 1 and 2. The concentration of 11-hydroxycorticisteroids, the major of which is corticosterone in rats, is considered an adaptive parameter and in accordance with the criteria (2) was used with positive sign while calculating the index. Plus and minus for each physiological or biochemical variable in Tables 2 and 4 do not mean the direction of changes but play technical (assistant) role and indicate the sign which should be assigned to the given parameter while calculating the sum.

In analyzing the bottom line in Table 2, one may conclude that trained animals (group *C*) are the most resistant to short-time intensive muscular load. Untrained and trained animals from the groups *D* and *E*, which were administered with eleutherosides, “rank” second and third, respectively. Intact rats from the group *B* proved to be least resistant to the above load. The most noteworthy fact is that the injection of eleutherosides simulates training and exerts a protective effect. This conclusion is evident from the comparison of *A* values for groups *B* and *D*.

## Example 2

### Materials and methods

Let us now consider one more example when the index of adaptedness was calculated for two groups of race horses differing in the functional status of the individuals [29]. The load intensity corresponded to about 80% of the maximum. This case takes into account recovery of variables 1 h after the test running (Tables 3 and 4). The perspiration, assessed visually and semi quantitatively, was not taken into analysis. The stability coefficients of other indices were found as the mean values of two magnitudes calculated separately for ${F}_1^{\prime }$ and ${F}_2^{\prime }$*.*

**Table 3:** Biochemical and physiological variables in horses before and after racing

Parameter	Group 1			Group 2		
	Baseline F° = F′	Running *F* ″	Recovery *F* ‴	Baseline *F° = F′*	Running *F* ″	Recovery *F* ‴
Blood oxygen release capacity (95% HbO_2_), μA	375 ± 14	393 ± 20	380 ± 16	330 ± 11	333 ± 9	325 ± 7
Blood oxygen release capacity (50% HbO_2_), μA	170 ± 5	185 ± 3	176 ± 19	163 ± 5	166 ± 4	161 ± 3
Blood Hb, g × 100 mL^− 1^	16.5 ± 0.2	16.9 ± 0.7	16.5 ± 0.2	15.1 ± 0.2	16.0 ± 0.5	15.3 ± 0.5
Heart rate, beat × min^− 1^	37.0 ± 1.5	112.9 ± 2.7	39.0 ± 2.9	42.5 ± 3.2	129.2 ± 2.9	45.0 ± 6.1
Blood lactate level, mg × 100 mL^− 1^	50.2 ± 28.9	55.5 ± 14.9	50.8 ± 7.1	59.0 ± 29.9	92.3 ± 13.5	64.2 ± 19.0
Reserve alkalinity of blood, mg × 100 mL^− 1^	550 ± 19	550 ± 26	560 ± 20	475 ± 22	430 ± 22	465 ± 11
Perspiration	No	++	+	No	+++	++

Values are expressed as Mean ± SD, *N* = 6 in each group. From Epstein and Rudoy [29]

**Table 4:** Calculation of the index of adaptedness of race horses to physical loads

Parameter	Group 1			Group 2		
	S	$\frac{\mid {K}^{{\prime\prime} }-{K}^{\prime}\mid }{K^{\prime }}$	$\frac{\mid {K}^{{\prime\prime\prime} }-{K}^{\prime}\mid }{\mid {K}^{{\prime\prime\prime} }-{K}^{{\prime\prime}}\mid }$	S	$\frac{\mid {K}^{{\prime\prime} }-{K}^{\prime}\mid }{K^{\prime }}$	$\frac{\mid {K}^{{\prime\prime\prime} }-{K}^{\prime}\mid }{\mid {K}^{{\prime\prime\prime} }-{K}^{{\prime\prime}}\mid }$
P_95_	0.963	+ 0.048	− 0.385	0.967	+ 0.009	− 0.625
P_50_	0.971	+ 0.088	−0.667	0.970	+ 0.018	−0.400
Hemoglobin	0.988	+ 0.024	−0	0.987	+ 0.059	−0.286
Heart rate	0.960	+ 2.059	−0.027	0.925	+ 2.040	−0.030
Lactate	0.424	−0.106	−0.128	0.493	−0.565	− 0.185
Blood reserve alkalinity	0.965	−0	−1.000	0.954	−0.095	−0.286
$A=\sum S\left(\left(\pm \right)\frac{\mid {K}^{{\prime\prime} }-{K}^{\hbox{'}}\mid }{K^{\hbox{'}}}-\frac{\mid K\hbox{'}\hbox{'}\hbox{'}-{K}^{\hbox{'}}\mid }{\mid {K}^{\hbox{'}\hbox{'}\hbox{'}}-{K}^{{\prime\prime}}\mid}\right)$		−0.094			−0.346	
Adaptive rank		1			2	

Increased pulse rate up to the definite limit, when indices of heart efficiency begin to decline, is undoubtedly an adaptive trait. Adaptive responses also include increased rate of oxygen release by the blood and increased concentration of hemoglobin.

### Results

In analyzing the changes in the variables studied, as well as the energetic efficiency of animals, Epstein and Rudoy [29] have characterized the horses from groups 1 and 2 as robust and weak, respectively. Thus, by means of precise quantitative calculations, the proposed method confirmed the conclusion of the above authors regarding the functional state if the animals from the groups compared.

## General discussion

In discussion, the following important notes should be made. The index of adaptedness *A* has no independent value whatsoever if not compared with the index of another group (or other groups) of organisms studied in parallel. According to the differentiation offered by Prosser and Brown [30], only regulating organisms, but not conformers, can be analyzed by this method. It is implied that the proposed algorithm can not be applicable to species of “poikilo-organisms”, or non-homeostatic animals, that are unable to maintain constant parameters of *milieu interieur.*

The calculation of SD for evaluating interindividual variability and hence functional stability makes sense for normally or at least symmetrically distributed variables. Therefore, non-Gaussian distributed parameters cannot be introduced in the proposed model. Further, it cannot operate parameters of nonlinear responses, particularly demonstrating the exponential dynamics during recovery process.

When referring to the topic, such terms as resistance, stability, tolerance, fitness, acclimation, coping, and adaptation are often equalized. This, however, is hardly justified, and, being a theoretical question, remains to be substantiated and should be the subject of a special study.

To be sure, the calculation of the suggested index of adaptedness cannot replace a detailed analysis of all the changes observed, since such an analysis is essential for elucidating specific mechanisms of adaptation.

## Conclusion

The model may be applied for assessing the functional state and resistance of athletes under different kinds of loads. It may also be used to study cross adaptation to two or several constraints, and in all experiments whose scheme may be represented by Fig. 1. A probable application of the method is the screening of pharmacological substances that one way or another affect the process of adaptation. Thus, the index of adaptedness makes it possible to quantify the integral response of bio systems to the action of disturbing factors. The said index is useful in that it replaces wordy descriptions of large tables that reveal alterations in numerous parameters of many of the subjects studied. The use of the model for analyzing experimental results would unavoidably force the investigator to take a more careful approach in designing experiment and in selecting the respective test groups, states and parameters. This, in turn, would lead to a more thorough methodological substantiation of the investigations in question.

## Data Availability

The program for calculating the index of adaptedness, designed in EXCEL computer package, for any experimental data and other materials are freely available from the author upon request.

## Abbreviations

11-HCS: 11-hydroxycorticosteroids
ATP: Adenosine triphosphate
FFA: Free fatty acids
P_50_: Blood oxygen release capacity at 50% HbO_2_
P_95_: Blood oxygen release capacity at 95% HbO_2_
SD: Standard deviation
